# Shorter long-term post-transplant life expectancy may be due to prior chemotherapy for the underlying disease: analysis of 3012 patients with acute myeloid leukemia enrolled on 9 consecutive ECOG-ACRIN trials

**DOI:** 10.1038/s41409-024-02308-0

**Published:** 2024-05-22

**Authors:** C. Ganzel, Y. Wang, K. Roopcharan, Z. Sun, J. M. Rowe, H. F. Fernandez, E. M. Paietta, S. M. Luger, H. M. Lazarus, L. D. Cripe, D. Douer, P. H. Wiernik, M. S. Tallman, M. R. Litzow

**Affiliations:** 1 Faculty of Medicine, Hebrew University of Jerusalem, Jerusalem, Israel; 2 Department of Hematology, Shaare Zedek Medical Center, Jerusalem, Israel; 3 Dana Farber Cancer Institute—ECOG-ACRIN Biostatistics Center, Boston, MA USA; 4 Moffitt Malignant Hematology & Cellular Therapy at Memorial Healthcare System, Pembroke Pines, USA; 5 Albert Einstein College of Medicine, New York, NY USA; 6 Division of Hematology/Oncology, Abramson Cancer Center, University of Pennsylvania, Philadelphia, PA USA; 7 Case Western Reserve University School of Medicine, Cleveland, OH USA; 8 Indiana University Simon Cancer Center, Indianapolis, IN USA; 9 Department of Hematology, University of Southern California Norris Comprehensive Cancer Center, Los Angeles, CA USA; 10 Cancer Research Foundation, Chappaqua, NY USA; 11 Memorial Sloan Kettering Cancer Center, New York, NY USA; 12 Division of Hematology, Mayo Clinic, Rochester, MN USA

**Keywords:** Haematopoietic system, Epidemiology

## Abstract

Several studies reported that patients with acute myeloid leukemia (AML) who remain in long-term remission after allogeneic or autologous transplant have a shorter life expectancy, compared to the general population. However, little is known about the life expectancy of adult long-term survivors of AML who were treated with chemotherapy alone without a transplant and there have been no comparisons with survival among the general population. The current study indicates that the life expectancy of AML patients who achieved and maintained CR for at least 3 years is shorter than expected for age in the US population. This was observed also in patients who did not undergo a transplant including those who have not relapsed during the entire long follow-up period. Thus, late relapse does not explain why patients without transplants have a shortened life expectancy. Taken together, these data strongly suggest that prior chemotherapy for the underlying AML is at least a major contributing factor for the known shortened life expectancy post-transplant.

## Introduction

The treatment of fit newly diagnosed patients with acute myeloid leukemia (AML) is based on intensive chemotherapy with or without hematopoietic cell transplantation (HCT). Allogeneic HSCT (allo-HCT), despite its potent anti-leukemic effect, also entails a great risk of early and late complications, partly related to graft-versus-host disease (GvHD). Thus, it is not surprising that patients with AML who remain in remission after allogeneic HCT have a shorter life expectancy, compared with the general population [1–5]. A similar finding, though less intuitive, was reported by the EBMT regarding autologous HCT (auto-HCT) [6]. Nevertheless, much less is known about the life expectancy of patients with AML who are treated with chemotherapy alone without undergoing a transplant. There are some data regarding pediatric patients with AML who were treated by chemotherapy alone [7, 8] but the data regarding adult patients are scarce [9, 10] and, to the best of our knowledge, a comparison of the survival of this patient population to the general (“normal”) population has not been reported. The question driving the current study is whether patients who are cured without transplant and survive 3 years in remission have a similar life expectancy to the sex- and age-matched normal population and, if not, could this at least partially explain the lower life expectancy observed post-transplant.

## Methods

### Study population

Between 1984 and 2008, 3012 patients, aged ≥15 years, with untreated AML were enrolled on nine consecutive phase II or III ECOG-ACRIN-led clinical trials (E1490, E1900, E3483, E3489, E3993, E3997, E3999, E4995, PC486) [11–19]. The individual protocol details are summarized in Table 1. The current analysis relates to patients who participated in these studies, reached complete remission (CR), and were relapse-free at 3 years. The analysis focused on the mortality trends, from the 3-year time period onward. Patients were divided into three groups based on the therapy received: 1) patients who underwent an allo-HCT, 2) patients who received an auto-HCT, and 3) patients who received only intensive chemotherapy and did not undergo any transplant (no transplant).

**Table 1:** AML protocols that were included in this study.

Protocol number (Study phase)	Induction	Consolidation	Activation-termination dates	Final accrual (Pts included)	Median follow-up year (95% CI)
E3483 [11] (III)	Dauno, ARAC, 6TG (DAT)	Matched sibling: alloBMT. Others: randomization to observation vs. maintenance vs. consolidation	Mar 1984–Jan 1988	534 (445)	14.79 (14.39, 15.16)
PC486 [12] (II)	DAT	Matched sibling: alloBMT Others: autoBMT	Apr 1987–Apr 1990	123 (98)	17.35 (15.37, 17.84)
E3489 [13] (III)	Idarubicin, ARAC	Idarubicin, ARAC HLA-matched or single-mismatched family member: alloBMT Others were randomized to: autoBMT vs ARAC	Feb 1990–Feb 1995	808 (753)	12.76 (12.53, 12.94)
E1490 [14] (III)	Dauno, ARAC, and GM-CSF vs. placebo	ARAC + GM-CSF vs. placebo	Sep 1990–Nov 1992	124 (115)	10.05 (9.08, 10.92)
E3993 [15] (III)	GM-CSF vs. placebo, ARAC Randomization: Dauno vs. Mitoxantrone vs. Idarubicin	Age < 70: ARAC + GM-CSF Age > 70: ARAC + GM-CSF	Apr 1993–Feb 1997	362 (343)	7.27 (5.66, 7.96)
E4995 [19] (II)	Dauno, ARAC	Age < 51 + HLA-matched sibling: alloPBSCT Others: ARAC, autoPBSCT	Aug 1996–Feb 1997	66 (59)	6.28 (5.87, 6.39)
E3997 [16] (II)	Dauno, ARAC + rhIL-11, GM-CSF	ARAC, rhIL-11, GM-CSF	June 1998–Apr 1999	36 (35)	8.02 (7.27, 8.25)
E3999 [17] (III)	Dauno, ARAC and zosuquidar vs. placebo	ARAC Dauno, ARAC and zosuquidar vs. placebo	Jul 2002–Sep 2005	449 (421)	4.96 (4.88, 4.99)
E1900 [18] (III)	Dauno 45 vs. 90, ARAC	Unfavorable/ intermediate risk or WBC > 100,000/µl + HLA-matched sibling: allo-HCT. Others: ARAC, GO vs. no GO, auto-HCT	Dec 2002–Nov 2008	657 (644)	8.28 (7.90, 8.65)

*allo* allogeneic, *ARAC* cytosine arabinoside, *auto* autologous, *BMT* bone marrow transplant, *dauno* daunorubicin, *GM-CSF* granulocyte-macrophage colony-stimulating factor, *GO* gemtuzumab ozogamicin, *HCT* hematopoietic cell transplant, *PB* peripheral blood, *6TG* 6-thioguanine.

### Cytogenetics

The definition of the different cytogenetic risk groups, based mostly on the published ECOG cytogenetic classification [20], is as follows:

Favorable: inv(16)/t(16;16)/del(16q) with or without other chromosome abnormalities; t(8;21) with or without additional abnormalities.

Intermediate: +8; −Y; +6; del(9q); del(12p); Normal karyotype.

Unfavorable: −5/del(5q); −7/del(7q); inv(3q)/t(3;3); Abnormal 20q or 21q; Translocation involving 11q23, t(6;9); t(9;22); Abnormal 17p; Complex karyotype defined as 3 or more abnormalities.

Indeterminate: All other clonal chromosomal abnormalities with less than three abnormalities, inconclusive cytogenetic studies, and any karyotype other than favorable, intermediate, and unfavorable karyotype listed above.

### Statistical considerations

Baseline characteristics of the patients in the three treatment groups were compared using Pearson’s chi-squared test if they were categorical and analysis of variance if they were continuous (Table 2).

**Table 2:** Characteristics of the patients, at diagnosis, according to the 3 groups of treatment (allo-HSCT, auto-HSCT, no transplant).

Variable	Value	Allogenic transplant	Autologous transplant	No transplant	Total	*P* value (Chi Sq)
*N*		78	110	315	503	
Age (years)	Mean (SD)	33.76 (10.66)	39.97 (11.88)	46.18(14.59)	42.9 (14.24)	<0.0001
	(Min, Max)	(16,54)	(18,60)	(15,79)	(15,79)	
Protocol	E1490	0	0	14 (100%)	14 (3%)	
	E1900	15 (11%)	57 (41%)	68 (49%)	140 (28%)	
	E3483	9 (13%)	0	59 (87%)	68 (14%)	
	E3489	51 (28%)	36 (20%)	92 (51%)	179 (36%)	
	E3993	0	0	13 (100%)	13 (3%)	
	E3997	0	0	7 (100%)	7 (1%)	
	E3999	0	0	34 (100%)	34 (7%)	
	E4995	0	0	23 (100%)	23 (5%)	
	PC486	3 (12%)	17 (68%)	5 (20%)	25 (5%)	
Sex	Male	34 (7%)	55 (11%)	149 (30%)	238 (47%)	0.686
	Female	44 (9%)	55 (11%)	166 (33%)	265 (53%)	
Performance status	0 or 1	71 (14%)	106 (21%)	280 (56%)	457 (91%)	0.064
	≥2	7 (1%)	4 (1%)	35 (7%)	46 (9%)	
Cytogenetics	Favorable	11 (2%)	29 (6%)	51 (10%)	91 (18%)	0.013
	Intermediate	34 (7%)	38 (8%)	108 (21%)	180 (36%)	
	Unfavorable	10 (2%)	2 (0%)	21 (4%)	33 (7%)	
	Indeterminate/missing	23 (5%)	41 (8%)	135 (27%)	199 (39%)	
White blood cell count (×10^**2**^**)**	*N*	78	110	315	503	0.163
	Mean (SD)	30.58 (50.8)	27.88 (34.38)	22.48 (36)	24.92 (38.39)	
	(Min, Max)	(0.6,366)	(0.2,178.9)	(0.3,360)	(0.2,366)	

Of the nine studies, only E1900, E3483, and E3489 included either an allo- or auto-HCT. Minimal cytogenetic information was available for patients enrolled on the earlier protocols (E1490, E3483, and PC486), which was consistent with the very limited cytogenetic data during this period.

**Fig. 1:**
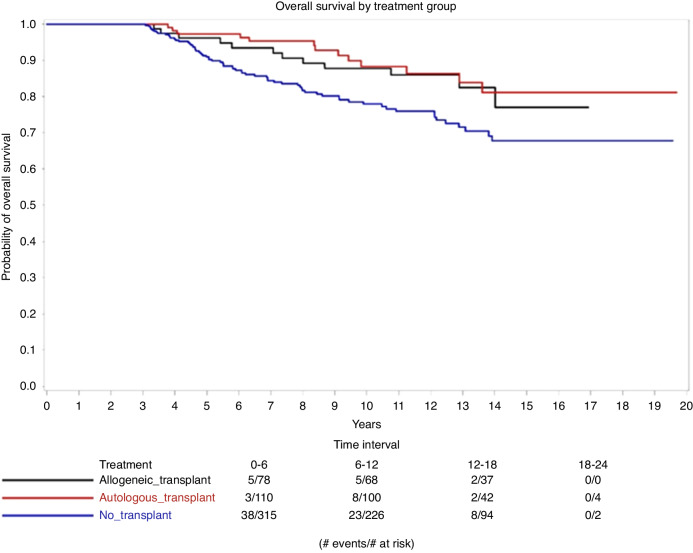
Overall survival by treatment group. A Cox proportional hazard regression model comparing the relationship between survival and treatment of patients who achieved CR and are disease-free at 3 years.

A Kaplan–Meier plot is shown in Fig. 1 considering death as an event and censoring for patients alive at last contact. The three curves were compared using a log-rank test under the null hypothesis that the hazard of death was the same for all three groups over the course of follow-up beyond the 3-year relapse-free time. Cox proportional hazard regression models stratified by protocol were generated for overall survival. In the multivariate model (Table 1-SUP.), age, hemoglobin, platelet and white blood cell counts, bone marrow and peripheral blood blast percentages, cytogenetics, and ECOG performance status were adjusted and compared using a chi-square test under the null hypothesis that the hazard of death was equivalent in each group.

### Observed versus expected mortality

Patients were grouped together by age and gender. They were matched to a normal population mortality rate for that age and gender group (Table 3A–C). The normal population comparator of *expected deaths* was derived using mortality rates from the Centers for Disease Control and Prevention (CDC) which reports life tables by year [21–23]. The specific table used for each treatment group (allo-HCT, auto-HCT, and no transplant) was determined by calculating the median year for that treatment group, which is the year when the midpoint of the total person years of follow-up in each treatment group occurred. Person years were calculated by tabulating the number of years lived in each age group for all subjects. Each year of follow-up for a patient with AML was counted as one person year, and the corresponding bin matched their age at the time (i.e., a subject who was diagnosed at age 26 years and survived until age 31 would contribute five person years to the 25–34 age group). Person years were then multiplied by the mortality rate of the matched normal population. This gives *expected deaths* for each age group and gender in each treatment group [2]. Chi-square statistics were derived using *expected deaths* and *observed deaths* and then summed for each gender and treatment, allowing for a test of the null hypothesis that patients with AML followed the mortality rate of the normal population given their age, gender, and treatment after surviving and remaining relapse-free at 3 years from diagnosis.

**Table 3:** Mortality tables.

A. Autologous transplant								
Age group	Sex	Number of subjects	Person years	Mortality rate	Expected deaths	Observed death	Chi-square	*P*-value
15–19	M	3	6	0.00091	0.005	0	0.005	0.94
20–24	M	9	34.7	0.00143	0.049	0	0.049	0.82
25–34	M	18	98.3	0.00145	0.142	1	5.163	0.02
35–44	M	24	134	0.00257	0.345	1	1.246	0.26
45–54	M	31	166.5	0.00552	0.919	1	0.007	0.93
55–64	M	24	140.9	0.0116	1.634	4	3.425	0.06
65–74	M	4	7.9	0.02738	0.217	0	0.217	0.64
15–19	F	1	2	0.00039	0.001	0	0.001	0.97
20–24	F	8	24	0.00050	0.012	0	0.012	0.91
25–34	F	16	92.9	0.00065	0.060	0	0.060	0.81
35–44	F	28	127	0.00149	0.189	1	3.482	0.06
45–54	F	33	203.2	0.00318	0.646	3	8.568	0.003
55–64	F	25	137	0.00730	1.001	0	1.001	0.32
65–74	F	8	18	0.01812	0.326	2	8.611	0.003
**B. Allogeneic Transplant**								
15–19	M	5	9	0.00124	0.011	0	0.011	0.92
20–24	M	11	42.5	0.00163	0.069	1	12.513	<0.001
25–34	M	23	129.8	0.00204	0.265	1	2.037	0.15
35–44	M	21	118.8	0.00327	0.389	3	17.531	<0.001
45–54	M	11	59.8	0.00591	0.353	0	0.353	0.55
55–64	M	6	22.2	0.01470	0.326	2	8.607	0.003
65–74	M	0	0	NA	NA	0	NA	NA
15–19	F	4	9.0	0.00045	0.004	0	0.004	0.95
20–24	F	9	35.0	0.00051	0.018	0	0.018	0.56
25–34	F	19	96.8	0.00075	0.073	0	0.073	0.79
35–44	F	26	156.9	0.00144	0.225	1	2.662	0.1
45–54	F	25	125.2	0.00328	0.411	3	16.326	<0.001
55–64	F	9	50.3	0.00858	0.431	1	0.750	0.39
65–74	F	3	7.5	0.01997	0.150	0	0.150	0.7
**C. No Transplant**								
15–19	M	5	13	0.00125	0.016	0	0.016	0.9
20–24	M	14	52	0.00161	0.084	0	0.084	0.77
25–34	M	29	158.6	0.00203	0.322	1	1.426	0.2
35–44	M	55	300.0	0.00332	0.995	6	25.192	<0.001
45–54	M	71	367.2	0.00593	2.176	8	15.585	<0.001
55–64	M	62	342.3	0.01432	4.901	11	7.589	0.006
65–74	M	38	127.0	0.03316	4.211	10	7.958	0.005
75–84	M	5	16.2	0.07458	1.210	1	0.036	0.85
15–19	F	5	10.3	0.00044	0.005	0	0.005	0.95
20–24	F	12	48.2	0.00051	0.024	1	38.963	<0.001
25–34	F	36	170.6	0.00076	0.130	3	63.239	<0.001
35–44	F	64	348.0	0.00146	0.508	2	4.387	0.036
45–54	F	73	411.9	0.00328	1.349	4	5.207	0.022
55–64	F	65	305.5	0.00838	2.560	10	21.621	<0.001
65–74	F	45	182.2	0.01983	3.613	10	11.294	<0.001
75–84	F	11	43.5	0.04824	2.097	2	0.0045	0.95

**Fig. 2:**
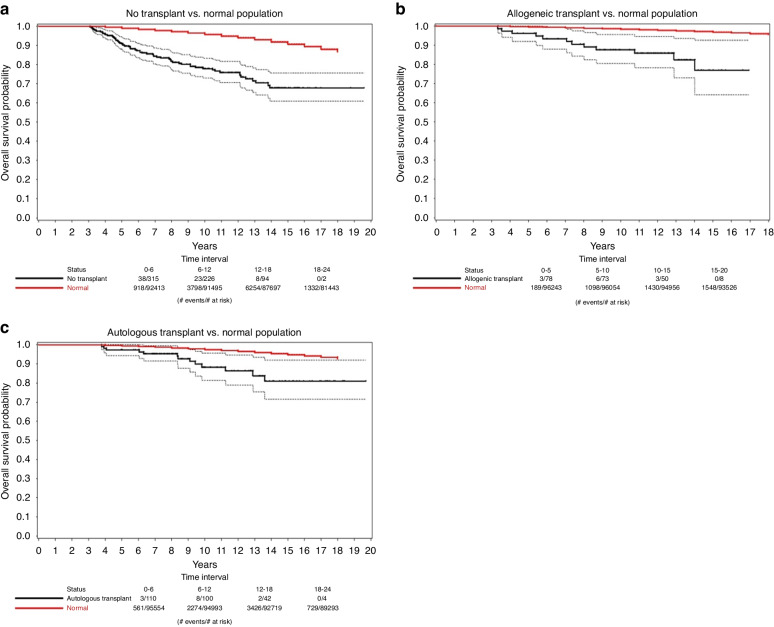
Overall survival of patients and normal population. Kaplan–Meier curve showing the survival probabilities of patients (black) against normal population (red). 95% confidence bands are represented by dashed lines. **a** No transplant (black) vs. normal population (red). **b** Allogenic transplant (black) vs. normal population (red). **c** Autologous transplant (black) vs. normal population (red).

The same US life tables from the CDC were used to get the survival of the matched normal population. The total population at the time of diagnosis for the normal population was set as the number of survivors in the life tables at the median age of diagnosis in the treatment group. The time of diagnosis was designated as year 1. The difference in survivor counts between each consecutive year and the preceding year was calculated to determine the number of deaths. Individuals who survived until year 19 were considered censored. Kaplan–Meier plots were used to compare overall survival to a matched US population (Fig. 2).

## Results

Among 3012 patients with newly diagnosed AML who were enrolled on nine consecutive phase II or III ECOG-ACRIN clinical trials, 503 achieved a CR and were relapse-free at 3 years. These patients with a mean age of 42.9 years old (range: 15–79) were included in the analysis; 78 patients underwent an allo-HCT; 110—auto-HCT and 315 did not undergo transplantation. The general median follow-up was 10.97 years and the median follow-up for each study is summarized in Table 1 which compares the nine clinical trials. Table 2 summarizes and compares the characteristics of the patients in the three treatment groups, at diagnosis. The mean age was different between the groups (33.76, 39.97, and 46.18 for the allo-HCT, auto-HCT, and no-transplant groups, respectively, *p* < 0.001), as well as platelet count (*p* = 0.03), WBC count (*p* = 0.02) and cytogenetics (*p* = 0.01). The percentage of intermediate cytogenetic risk, for example, was only 7–8% among the allo- and auto-HCT patients and 21% among the no-transplant patients. The 10-year OS was 87.75% for the allo-HCT group, 88.28% for the auto-HCT group, and 77.97% for the no-transplant group. Although in the Kaplan–Meier curve (Fig. 1) the autologous transplant group had a decreased hazard of death when compared with the no transplant group, the multivariate Cox proportional hazard regression model presented non statistically significant different hazards for the two groups (HR (95%CI) = 0.63(0.30,1.32), *p* = 0.22, Table 1-SUP.).

Table 3 shows the life table for each of the 3 treatment groups. The CDC life tables were chosen according to the median year of each group, which was 2003, 1993, and 1994 for the auto-HCT, allo-HCT, and no transplant groups. As an example, patients from the no-HCT group (Table 3c), who are males in the age 35–44 (*n* = 55) were compared to the same age and gender persons in the matched CDC life table. The mortality rate for that group according to the CDC life table is 0.0332, so the expected death is 0.995 but the observed death among the group of no-transplant patients was six. The difference between the expected and observed death was statistically significant (*p* < 0.001). In every treatment group, patients had shorter survival compared with their age- and the gender-matched general population (*p*-value of 0.004 for the auto-HSCT group and <0.001 for both allo-HSCT and non-transplant groups).

Figure 2a–c compares the overall survival probability of the three treatment cohorts to the matched US normal populations. All three treatment groups have decreased survival probabilities compared with the matched normal population. In other words, patients with AML who have survived relapse-free for at least 3 years continue to have a greater mortality rate when compared with the normal population for up to 14 years.

**Fig. 3:**
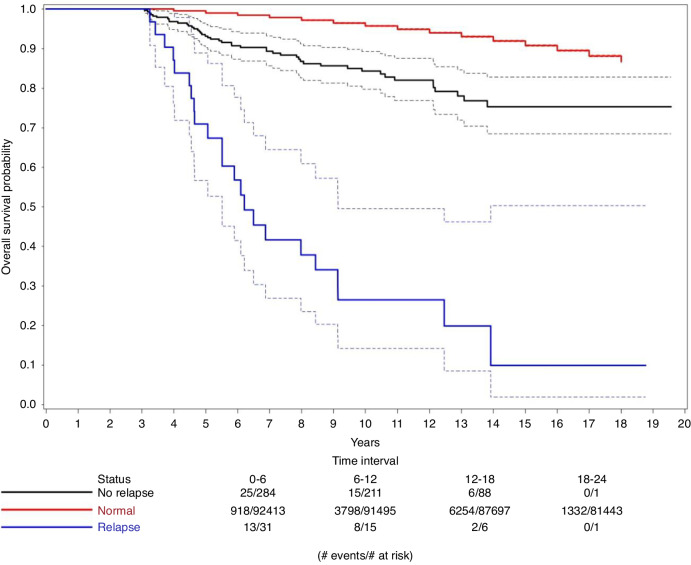
Overall survival of no-transplant patients by relapse status. Kaplan–Meier curve showing the survival probabilities of no-transplant patients who relapsed beyond 3 years (blue) and no-transplant patients who did not relapse (black) against normal population (red). 95% confidence bands are represented by dashed lines.

**Fig. 4:**
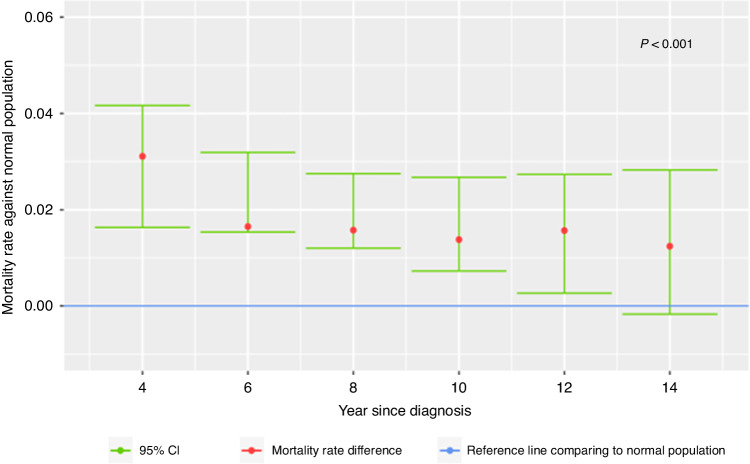
Mortality rate of patients who did not undergo a transplant and have not relapsed over the matched normal population. The blue line represents the normal population. The red dots with their 95% confidence interval (in green) represent the mortality rate difference from the normal population. This figure demonstrates graphically that even patients who did not undergo a transplant and have not relapsed during the years of follow-up have a higher mortality rate compared to the normal population.

Finally and crucially, it is important to show that relapse is not the only reason for the decreased life expectancy among the no-transplant patients. At the general median follow-up of 10.97 years, 35 relapses were observed, 5.7% (*n* = 2) were from the allo-HCT group, 8.6% (*n* = 3) from the auto-HCT and 85.7% (*n* = 30) from the no transplant group. Figure 3 shows the survival curves of the no transplant patients who relapsed beyond 3 years and of those who have not relapsed and compares them to the normal population. Both groups have decreased survival probabilities compared with the normal population. Figure 4 demonstrates that the mortality rate of patients who have not undergone transplant and have not relapsed during the entire follow-up period is still significantly greater than that of the normal population.

## Discussion

It has long been established that the long-term life expectancy of patients undergoing an allogeneic HCT is lower than may be expected from a comparable “normal” population. This includes patients thought to be cured without evidence for any active chronic GvHD. While theories abound, a prevailing assumption is that this is in some way related to the inherent immunological reset that follows a successful allogeneic transplant [5, 24]. Such an assumption would not explain why a similar observation was reported following an autologous transplant. The possibility that factors unrelated to the transplant itself, such as the intensive chemotherapy given for the AML prior to the transplant, may contribute to these observations, formed the hypothesis for this study. To explore this hypothesis a detailed analysis was initiated to explore the actual life expectancy of patients with AML who did not receive any transplant and are ‘cured’ of their disease. Data from nine consecutive studies from the ECOG-ACRIN were used for this analysis.

The focus was on patients who received treatment for AML, entered a CR, and had not relapsed for 3 years. The data demonstrated that among the group of patients who did not have a transplant, the mortality rate was significantly higher than that of a comparable normal population, as determined from life tables of the CDC. The possible reasons for the higher mortality rate of patients who received only chemotherapy need to be elucidated. Although the rate of late relapse in AML is reported to be low [25, 26], such recurrences do occur and clearly may contribute to the increased mortality. In addition, late effects of chemotherapy [27], such as secondary malignancies [28], could explain part of the picture. Other predisposing factors, such as clonal hematopoiesis of indeterminate potential (CHIP) may increase the morbidity of different diseases such as atherosclerotic cardiovascular disease [29], cerebrovascular accidents (CVA) [30], and COPD [31]. In addition, other organ toxicities (cardiac, renal, or hepatic) may also increase the mortality rate. The precise cause of death in the ECOG-ACRIN clinical trials that formed the basis of this study was only captured in the very recent trials. It was thus important to demonstrate that relapse in and of itself cannot explain the entire picture. This led to the focus on the subgroup of patients who survived in CR for three years and have not relapsed throughout the entire long follow-up. This analysis demonstrated that even this selected cohort of patients has a higher mortality rate compared with the normal population.

Although the survival of patients with AML has changed over the period of this study, the cohorts of interest, after at least three years in CR, are likely to have a similar follow-up care. An analysis of each original study, by age and year, comprised tiny cohorts of patients, confounding any meaningful interpretation. At the same time, it remains entirely speculative whether advances in supportive care, such as vaccination strategies, anti-microbial prophylaxis, or improved cardiovascular screening, may impact the overall data in a group of patients spanning three decades.

Patients included in this analysis were treated over a period of time where there has been a marked evolution in transplantation practice and supportive care. Nevertheless, it seems likely that the issue remains relevant also in contemporary strategies [32]. Patients selected in this study are those who entered CR1 and maintained this CR for at least 3 years. We are not aware of any data that the biological likelihood of late relapse beyond 3 years, for those who were not transplanted and remained in CR throughout this period, has changed over the years (1984–2008) depending on the type of therapy that was used. It was therefore felt that selecting a larger population cohort, even if spread over more years, is reasonable and preferable to breaking this down into smaller groups with data that would be difficult to interpret.

In conclusion, the life expectancy of patients with AML who achieve and maintain CR for at least 3 years is shorter compared with the normal US population. This applies to patients who had a transplant, which has been known for a long time, but also to patients who did not have a transplant. For patients who were not transplanted, the reason for the shortened life expectancy is unknown but cannot be explained solely by late relapse. The etiology for the shortened life expectancy post HCT, after allowing for all known transplant complications and sequelae, has been a challenge. The data presented herein suggest at least a major contributing factor.

### Supplementary information

Table 1-Sup

## Data Availability

All data generated or analysed during this study are included in this published article and its supplementary information files.
