# Correction: Shorter long-term post-transplant life expectancy may be due to prior chemotherapy for the underlying disease: analysis of 3012 patients with acute myeloid leukemia enrolled on 9 consecutive ECOG-ACRIN trials

**DOI:** 10.1038/s41409-024-02337-9

**Published:** 2024-07-18

**Authors:** C. Ganzel, Y. Wang, K. Roopcharan, Z. Sun, J. M. Rowe, H. F. Fernandez, E. M. Paietta, S. M. Luger, H. M. Lazarus, L. D. Cripe, D. Douer, P. H. Wiernik, M. S. Tallman, M. R. Litzow

**Affiliations:** 1 Faculty of Medicine, Hebrew University of Jerusalem, Jerusalem, Israel; 2 Department of Hematology, Shaare Zedek Medical Center, Jerusalem, Israel; 3 Dana Farber Cancer Institute—ECOG-ACRIN Biostatistics Center, Boston, MA USA; 4 Moffitt Malignant Hematology & Cellular Therapy at Memorial Healthcare System, Pembroke Pines, FL USA; 5 Albert Einstein College of Medicine, New York, NY USA; 6 Division of Hematology/Oncology, Abramson Cancer Center, University of Pennsylvania, Philadelphia, PA USA; 7 Case Western Reserve University School of Medicine, Cleveland, OH USA; 8 Indiana University Simon Cancer Center, Indianapolis, IN USA; 9 Department of Hematology, University of Southern California Norris Comprehensive Cancer Center, Los Angeles, CA USA; 10 Cancer Research Foundation, Chappaqua, NY USA; 11 Memorial Sloan Kettering Cancer Center, New York, NY USA; 12 Division of Hematology, Mayo Clinic, Rochester, MN USA

**Keywords:** Haematopoietic system, Epidemiology

Correction to: *Bone Marrow Transplantation* 10.1038/s41409-024-02308-0, published online 22 May 2024

In this article, the author’s name Yating Wang was incorrectly written as Wang Yating.

The original article has been corrected.

