# Supplementary material for: Shorter long-term post-transplant life expectancy may be due to prior chemotherapy for the underlying disease: analysis of 3012 patients with acute myeloid leukemia enrolled on 9 consecutive ECOG-ACRIN trials
Source: Bone Marrow Transplant. 2024 May 22;59(9):1215–23. doi: 10.1038/s41409-024-02308-0 (PMC11368814; doi:10.1038/s41409-024-02308-0)
Supplement: Supplementary file 1 — Table 1-Sup [file 41409_2024_2308_MOESM1_ESM.docx]

**Table 1-SUP.** Multivariate model


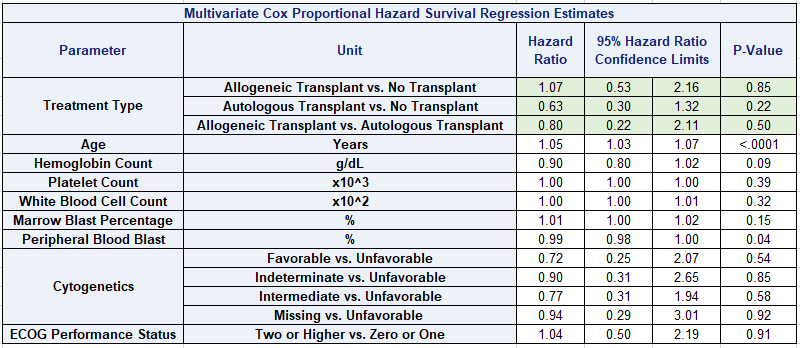


**Table 1-SUP.** A cox proportional hazard regression model was generated comparing the relationship between survival and treatment while adjusting for age, hemoglobin, platelet, and white blood cell counts, bone marrow and peripheral blood blast percentage, cytogenetics, and ECOG performance status. For both allogenic and autologous HSCT’s the null hypothesis that the hazard of death for either group when compared with receiving no transplant is equivalent fails to be rejected (two tailed, alpha of 0.05). It seems that the hazard of death is the same irrespective of treatment group.
